# Crystal structure of N-terminally hexahistidine-tagged *Onchocerca volvulus* macrophage migration inhibitory factor-1

**DOI:** 10.1107/S2053230X24010550

**Published:** 2024-11-06

**Authors:** Amber D. Kimble, Omolara C. O. Dawson, Lijun Liu, Sandhya Subramanian, Anne Cooper, Kevin Battaile, Justin Craig, Elizabeth Harmon, Peter Myler, Scott Lovell, Oluwatoyin A. Asojo

**Affiliations:** a Department of Clinical Laboratory Science, College of Nursing and Allied Health Sciences Howard University 801 North Capitol Street, 4th Floor Washington DC20002 USA; b Grafton High School USA, 403 Grafton Drive, Yorktown, VA23692, USA; c Protein Structure and X-ray Crystallography Laboratory University of Kansas 2034 Becker Drive Lawrence KS66047 USA; d Seattle Structural Genomics Center for Infectious Diseases, Seattle, Washington, USA; e Center for Global Infectious Disease Research Seattle Children’s Research Institute 307 Westlake Avenue, North Suite 500 Seattle WA98109 USA; f NYX, New York Structural Biology Center, Upton, NY11973, USA; g University of Kansas 2034 Becker Drive Lawrence KS66218 USA; h Dartmouth Cancer Center, One Medical Center Drive, Lebanon, NH03756, USA; University of York, United Kingdom

**Keywords:** Seattle Structural Genomics Center for Infectious Disease, macrophage migration inhibitory factor-1, river blindness, onchocerciasis, nodding syndrome

## Abstract

*Onchocerca volvulus* causes blindness, onchocerciasis, skin infections and devastating neurological diseases such as nodding syndrome. New treatments are needed because the currently used drug, ivermectin, is contraindicated in pregnant women and those co-infected with *Loa loa*. The Seattle Structural Genomics Center for Infectious Disease (SSGCID) produced, crystallized and determined the apo structure of N-terminally hexahistidine-tagged *O. volvulus* macrophage migration inhibitory factor-1 (His-*Ov*MIF-1). *Ov*MIF-1 is a possible drug target. His-*Ov*MIF-1 has a unique jellyfish-like structure with a prototypical macrophage migration inhibitory factor (MIF) trimer as the ‘head’ and a unique C-terminal ‘tail’. Deleting the N-terminal tag reveals an *Ov*MIF-1 structure with a larger cavity than that observed in human MIF that can be targeted for drug repurposing and discovery. Removal of the tag will be necessary to determine the actual biological oligomer of *Ov*MIF-1 because size-exclusion chomatographic analysis of His-*Ov*MIF-1 suggests a monomer, while *PISA* analysis suggests a hexamer stabilized by the unique C-terminal tails.

## Abstract 2

N-terminally hexahistidine-tagged *O. volvulus* macrophage migration inhibitory factor-1 has a unique jellyfish-like structure with the prototypical macrophage migration inhibitory factor trimer as the ‘head’ and a C-terminal extension as the ‘tail’.

## 1. Introduction

The filarial nematode *Onchocerca volvulus* causes onchocerciasis or river blindness, the second leading cause of global blindness and a neglected disease with a global disease burden of 1.23 million disability-adjusted life years (Tirados *et al.*, 2022 ▸). *O. volvulus* is transmitted to humans by blackflies. In addition to causing blindness and severe eye and skin diseases, *O. volvulus* is associated with ‘nodding syndrome’, a serious neurological disorder (Colebunders *et al.*, 2023 ▸). Nodding syndrome has been reported in Tanzania, northern Uganda and South Sudan, affecting children aged 5–15 years and resulting in progressive neurological and intellectual decline, stunted growth and characteristic immunologic epilepsy (Colebunders *et al.*, 2023 ▸; Olum *et al.*, 2020 ▸; Benedek *et al.*, 2020 ▸). Additionally, onchocerciasis causes premature mortality and morbidity and increased child mortality rates from high disease burden and onchocerciasis-related epilepsy (Van Cutsem *et al.*, 2024 ▸; Olum *et al.*, 2020 ▸; Chesnais *et al.*, 2018 ▸).

Onchocerciasis is currently controlled with hygiene efforts and school-based mass drug administration of the antiparasitic drug ivermectin (Martin *et al.*, 2021 ▸; Colebunders *et al.*, 2024 ▸; Stapley *et al.*, 2024 ▸). Ivermectin is an inexpensive drug that effectively eradicates the parasite’s dermal stage (microfilariae), preventing skin damage and vision loss (Cupp *et al.*, 2011 ▸; Nikièma *et al.*, 2018 ▸; Higazi *et al.*, 2014 ▸). Between 1988 and 2009, >800 million ivermectin doses were administered, significantly diminishing the prevalence of onchocerciasis in more than 25 countries and disrupting its transmission in ten countries (Cupp *et al.*, 2011 ▸; Tekle *et al.*, 2012 ▸). Alternative therapeutic approaches are needed because ivermectin cannot eradicate adult worms, so the transmission cycle continues (Cupp *et al.*, 2011 ▸; Erber *et al.*, 2021 ▸). *O. volvulus* eradication efforts are hampered by adverse reactions to ivermectin, including death, in patients who are co-infected with high levels of the nematode parasite *Loa loa* (Boullé *et al.*, 2023 ▸).

Ivermectin is contraindicated in treating pregnant women because ivermectin is a known mammalian teratogen (Erber *et al.*, 2021 ▸; Nicolas *et al.*, 2020 ▸). The historical exclusion of pregnant women from community-based drug (ivermectin) administration programs leads to heightened maternal and infant mortality, with implications for future ‘parasitic tolerance’ in prenatally exposed children (Nicolas *et al.*, 2020 ▸; Colebunders *et al.*, 2024 ▸). Furthermore, repeated ivermectin treatments may genetically select treatment-resistant adult female worms, thus negating the anti-fecundity effects of ivermectin (Tirados *et al.*, 2022 ▸; Lustigman & McCarter, 2007 ▸; Osei-Atweneboana *et al.*, 2011 ▸).

*O. volvulus* and other parasitic nematodes co-evolved with their hosts and are experts at surviving in hostile microenvironments, where they feed on host blood and release molecules such as macrophage migration inhibitory factor (MIF) homologs to evade or suppress host immune responses for several years without killing the host (Loukas *et al.*, 2005 ▸; Loukas & Prociv, 2001 ▸; Rodríguez-Sosa *et al.*, 2003 ▸; Vermeire *et al.*, 2008 ▸). Human MIF (hMIF) is a cytokine with tautomerase activity that binds the CD74 receptor (Cho *et al.*, 2011 ▸; Vermeire *et al.*, 2008 ▸). CD74 binding is necessary for the roles of hMIF in recruiting immune cells and activating cellular responses, including cell proliferation and metabolism (Bernhagen *et al.*, 2007 ▸; Klasen *et al.*, 2014 ▸). Parasite MIF homologs are vital for the survival and proliferation of these parasites in the host (Cho *et al.*, 2011 ▸; Vermeire *et al.*, 2008 ▸). Targeting parasite MIF orthologues offers alternatives for drug development for neglected tropical diseases since some parasite MIF inhibitors have been shown to kill the parasites selectively (Cho *et al.*, 2007 ▸).

*O. volvulus* and many parasitic nematodes secrete MIF homologs to facilitate evasion of the host’s immune system (Rodríguez-Sosa *et al.*, 2003 ▸; Vermeire *et al.*, 2008 ▸). Parasite MIFs may facilitate immune evasion by altering macrophage influx, immune cell activation and host cytokine production (Falcone *et al.*, 2001 ▸). Like their mammalian MIF counterparts, parasite MIF homologs inhibit host macrophage migration, and neutralizing antibodies can abort the inhibition (Vermeire *et al.*, 2008 ▸; Pastrana *et al.*, 1998 ▸). Parasite MIFs have tautomerase activity and catalyze the keto–enol isomerization of aromatic substrates such as hydroxyphenyl­pyruvate and l-dopachrome methyl ester (Vermeire *et al.*, 2008 ▸). To clarify the structural basis of these functions, we produced, crystallized and determined the 1.9 Å resolution structure of N-terminally hexahistidine-tagged macrophage migration inhibitory factor-1 (His-*Ov*MIF-1).

## 2. Materials and methods

### 2.1. Macromolecule production

His-*Ov*MIF-1 was cloned, expressed and purified using established protocols (Stacy *et al.*, 2011 ▸; Serbzhinskiy *et al.*, 2015 ▸; Rodríguez-Hernández *et al.*, 2023 ▸; Buchko *et al.*, 2013 ▸). The full-length gene for macrophage migration inhibitory factor-1 from *O. volvulus* (UniProt Q963F7) encoding amino acids 1–115 was codon-optimized for expression in *Escherichia coli* and synthesized by the gene-synthesis vendor Twist Bioscience. The codon-optimized sequence was ligated into the expression vector pET-28a to generate plasmid DNA. The final design incorporates a hexahistidine and 3C protease cleavage site for cleavage between TQ/GPGS that forms the N-terminal extension (Table 1 ▸).

Plasmid DNA was transformed into chemically competent *E. coli* BL21(DE3)R3 Rosetta cells. The plasmid containing His-*Ov*MIF-1 was tested for expression and 2 l of culture was grown using auto-induction medium (Studier, 2005 ▸) in a LEX Bioreactor (Epiphyte Three) as described previously (Serbzhinskiy *et al.*, 2015 ▸). The expression clone can be requested online at https://www.ssgcid.org/available-materials/expression-clones/.

His-*Ov*MIF-1 was purified in two steps: an immobilized metal (Ni^2+^) affinity chromatography (IMAC) step and size-exclusion chromatography (SEC) on an AKTApurifier 10 (GE Healthcare) using automated IMAC and SEC programs (Serbzhinskiy *et al.*, 2015 ▸). Briefly, thawed bacterial pellets (25 g) were lysed by sonication in 200 ml lysis buffer [25 m*M* HEPES pH 7.0, 500 m*M* NaCl, 5%(*v*/*v*) glycerol, 0.5%(*w*/*v*) CHAPS, 30 m*M* imidazole, 10 m*M* MgCl_2_, 1 m*M* TCEP, 250 mg ml^−1^ AEBSF, 0.025%(*w*/*v*) sodium azide]. After sonication, nucleic acids were degraded from the crude lysate by adding 20 µl (25 units ml^−1^) of Benzonase and incubating while mixing at room temperature for 45 min. The lysate was clarified by centrifugation at 10 000 rev min^−1^ for 1 h using a Sorvall centrifuge (Thermo Scientific). The clarified supernatant was then passed over an Ni–NTA HisTrap FF 5 ml column (GE Healthcare) which had been pre-equilibrated with loading buffer [25 m*M* HEPES pH 7.0, 500 m*M* NaCl, 5%(*v*/*v*) glycerol, 30 m*M* imidazole, 1 m*M* TCEP, 0.025%(*w*/*v*) sodium azide]. The column was washed with 20 column volumes (CV) of loading buffer and eluted with elution buffer [25 m*M* HEPES pH 7.0, 500 m*M* NaCl, 5%(*v*/*v*) glycerol, 30 m*M* imidazole, 1 m*M* TCEP, 0.025%(*w*/*v*) sodium azide, 250 m*M* imidazole] over a 7 CV linear gradient. Peak fractions were pooled, concentrated to 5 ml and loaded onto a Superdex 75 column (GE Healthcare) equilibrated with running buffer (25 m*M* HEPES pH 7.0, 500 m*M* NaCl, 5% glycerol, 2 m*M* DTT, 0.025% azide). His-*Ov*MIF-1 eluted as a single/monodisperse peak at ∼19 kDa, indicating a monomer. The peak fractions were collected and analyzed using a reduced SDS–PAGE gel. Pure peak fractions were pooled and concentrated to 20 mg ml^−1^ using an Amicon purification system (Millipore). Aliquots of 110 µl were flash-frozen in liquid nitrogen and stored at −80°C until use. Purified His-*Ov*MIF-1 can be requested online at https://www.ssgcid.org/available-materials/ssgcid-proteins/.

### 2.2. Crystallization

Purified His-*Ov*MIF-1 includes an N-terminal extension (hexahistidine and 3C protease site; underlined in Table 1 ▸), which was not removed before crystallization. His-*Ov*MIF-1 was crystallized at 291 K using sitting-drop vapor diffusion. Briefly, 20.6 mg ml^−1^ protein was mixed with precipitant solution in a 1:1 ratio as described in Table 2 ▸. The protein buffer was 5 m*M* HEPES pH 7.0, 500 m*M* NaCl, 5% glycerol, 2 m*M* DTT, 0.025% azide, while the precipitant solution was 100 m*M* bis-Tris–HCl pH 6.5, 400 m*M* sodium chloride, 30% (*w*/*v*) PEG 3350. No additional cryoprotectant was added before flash-cooling for data collection.

### 2.3. Data collection and processing

Table 3 ▸ describes the data collection. The data were integrated with *XDS* (Kabsch, 2010 ▸) and reduced with *AIMLESS* (Evans, 2006 ▸). Raw X-ray diffraction images have been stored at the Integrated Resource for Reproducibility in Macromolecular Crystallography (https://proteindiffraction.org/project/OnvoA_00834_a_UX1-Apo_8vj2/).

### 2.4. Structure solution and refinement

The structure of His-*Ov*MIF-1 was determined by molecular replacement with *Phaser* (McCoy *et al.*, 2007 ▸) from the *CCP*4 suite of programs (Collaborative Computational Project, Number 4, 1994 ▸; Krissinel *et al.*, 2004 ▸; Winn *et al.*, 2011 ▸; Agirre *et al.*, 2023 ▸) using PDB entry 1mif (Sun *et al.*, 1996 ▸) as the search model. Structure refinement and manual model building were carried out with *Phenix* (Liebschner *et al.*, 2019 ▸) and *Coot* (Emsley *et al.*, 2010 ▸), respectively. The structure quality was checked using *MolProbity* (Williams *et al.*, 2018 ▸). Data-reduction and refinement statistics are shown in Table 4 ▸. Coordinate and structure factors have been deposited with the Worldwide PDB (wwPDB) as entry 8vj2. The structure was inspected with the *CheckMyBlob* server, which identified no additional blobs or ligands (https://checkmyblob.bioreproducibility.org/server/; Kowiel *et al.*, 2019 ▸).

## 3. Results and discussion

**Figure 1:**
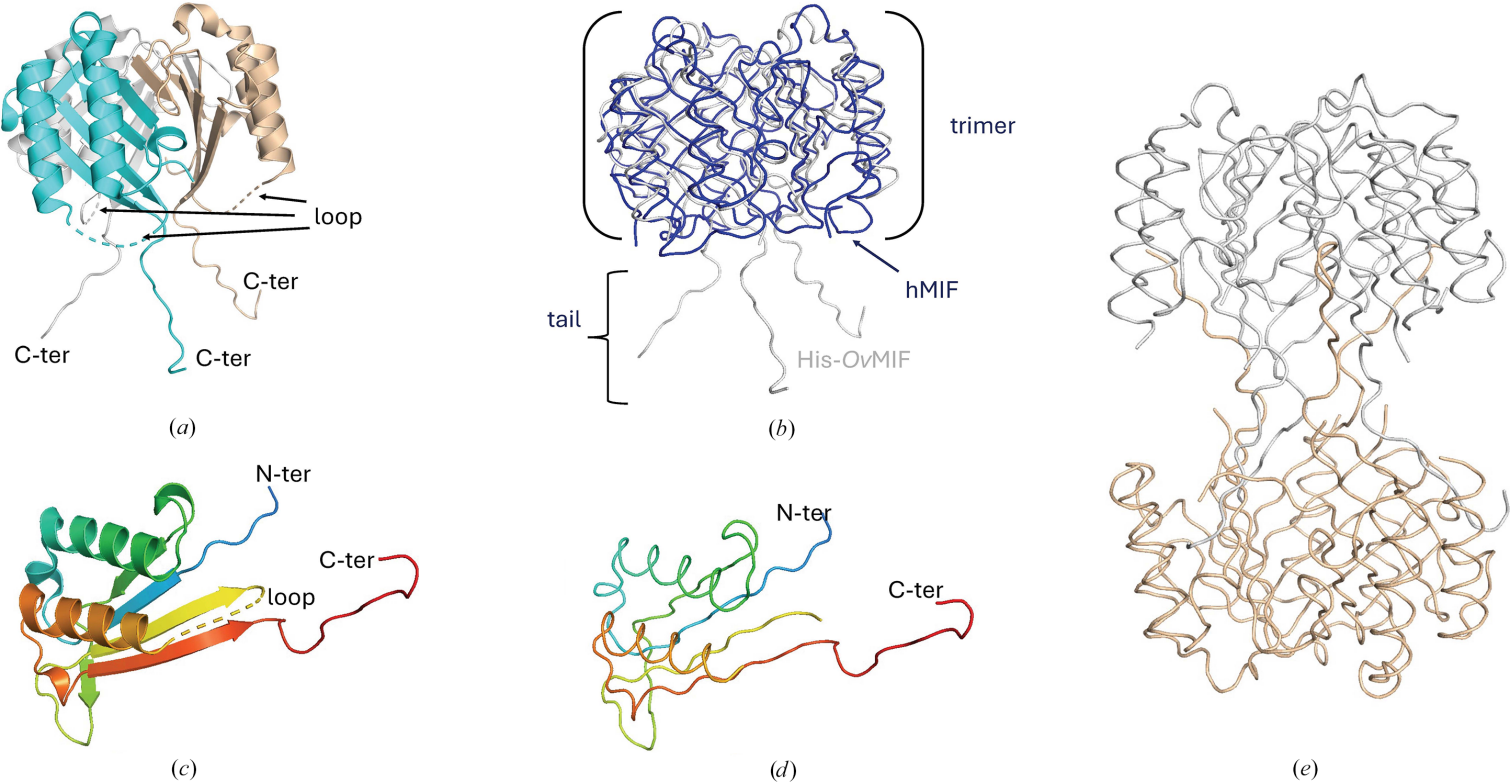
His-*Ov*MIF-1 structure. (*a*) His-*Ov*MIF-1 trimer with monomers colored cyan, wheat and gray. (*b*) The His-*Ov*MIF-1 trimer (gray) has a jellyfish-like structure and the head aligns with the prototypical hMIF (blue). (*c*) Cartoon diagram of the longest His-*Ov*MIF-1 monomer colored in a rainbow from blue at the N-terminus to red at the C-terminus. (*d*) Ribbon diagram of the longest His-*Ov*MIF-1 monomer colored in a rainbow from blue at the N-terminus to red at the C-terminus. (*e*) *PISA*-generated His-*Ov*MIF-1 tetramer.

The 1.9 Å resolution apo structure of His-*Ov*MIF-1 is reported. SEC data indicate that the protein purifies as a monomer. There are three His-*Ov*MIF-1 monomers in the asymmetric unit (Fig. 1 ▸*a*). Unlike the molecular-replacement search model (PDB entry 1mif; human MIF, hMIF-1), His-*Ov*MIF-1 has a unique C-terminal tail that extends outwards and makes the structure resemble a jellyfish (Fig. 1 ▸*b*). The quality of the electron-density maps for the structure is shown in Supplementary Fig. S1. *Proteins, Interfaces, Structures and Assemblies* (*PISA*) analysis (Krissinel, 2010 ▸; Krissinel & Henrick, 2005 ▸, 2007 ▸) suggests that the most stable assembly is a hexamer in which the tails form intermolecular β-strands (Fig. 1 ▸*e*). The hexamer has a dumbbell shape, with each head of the dumbbell consisting of the classical MIF trimer (Supplementary Fig. S2 and Fig. 1 ▸*e*). The hexamer is more stable than the trimer based on its calculated Δ*G*^diss^ value of 72.5 kcal mol^−1^ compared with 11.5 kcal mol^−1^ (Supplementary Fig. S2) for the trimer. Further analysis of untagged *Ov*MIF-1 is required to determine the biological unit.

**Figure 2:**
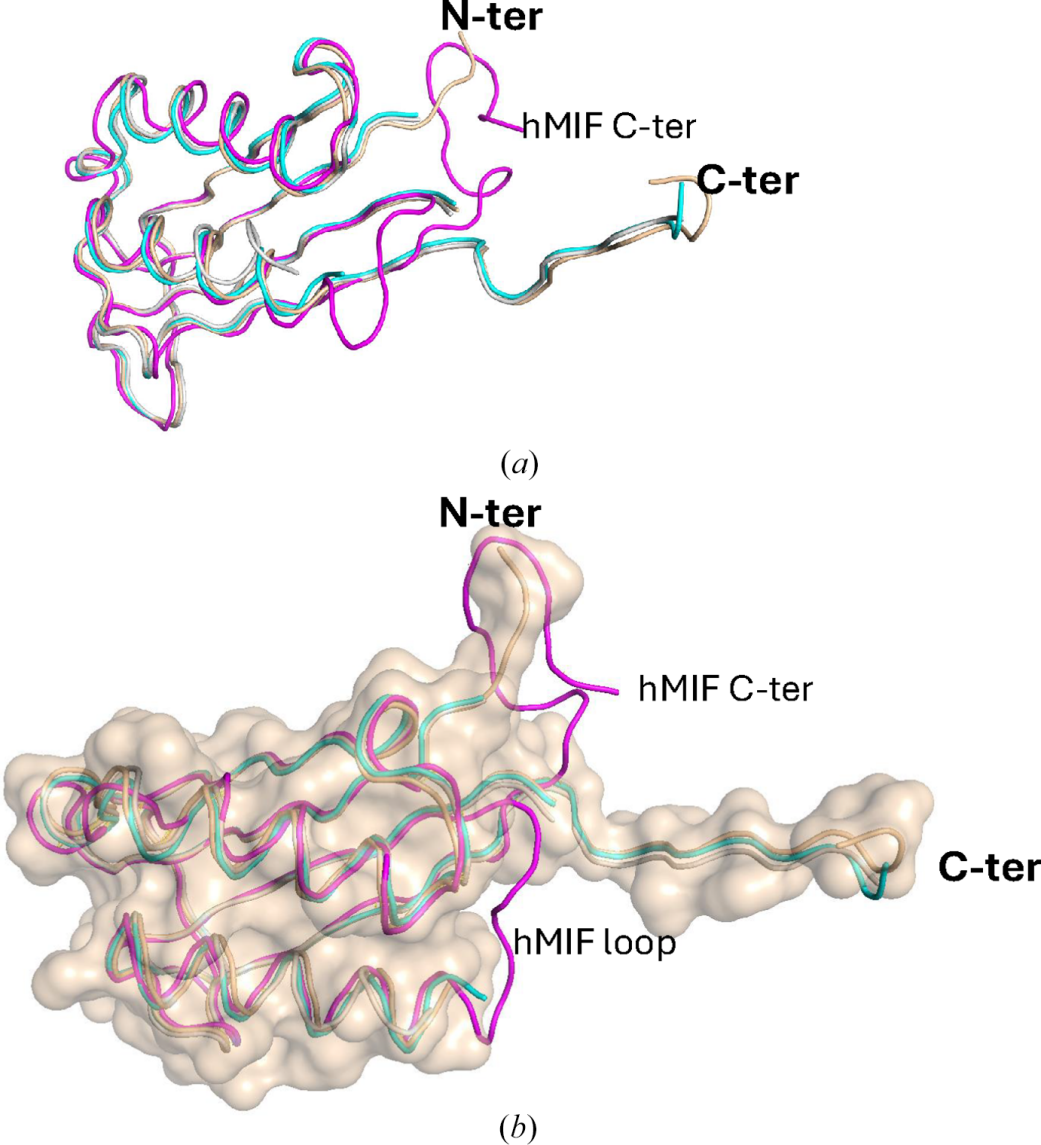
Comparison of the His-*Ov*MIF-1 and hMIF structures. (*a*) The superposed His-*Ov*MIF-1 monomers (colored cyan, wheat and gray) are very similar. hMIF (colored magenta) lacks the tail. (*b*) The surface of His-*Ov*MIF-1 (wheat) shows how its N-terminus occupies the location of the C-terminal residues of hMIF.

Two chains have 111 ordered amino acids and the third has 105 of the 115 main-chain amino acids of His-*Ov*MIF-1. Loop Cys67–Asn71 is disordered in all three monomers, and the N-terminal hexahistidine tag is disordered in all monomers. The three monomers are similar (Fig. 2 ▸*a*), with an r.m.s.d. of 0.36 Å for the main-chain carbons (100 residues aligned). A closer comparison of the His-*Ov*MIF-1 structures with hMIF-1 reveals that the additional N-terminal amino acids of His-*Ov*MIF-1 block the space occupied by the carboxyl-terminus of hMIF-1 (Fig. 2 ▸*b*) and prevent its ability to fold back, and may account for the formation of the unique ‘tail’ instead. *PDBeFold* (https://www.ebi.ac.uk/msd-srv/ssm/; Krissinel & Henrick, 2004 ▸) analysis using the default threshold of 70% was used to identify the nearest structural neighbors of His-*Ov*MIF-1 (Supplementary Table S2). Interestingly, the closest structural neighbors were hMIF structures, which was validated by *ENDScript* (Gouet *et al.*, 2003 ▸; Robert & Gouet, 2014 ▸) analysis (Supplementary Fig. S3). His-*Ov*MIF-1 has a prototypical MIF topology, except for the C-terminal extension and regions near the N-terminus, which are thicker in the *ENDScript* sausage plot (Supplementary Fig. S3). There is no correlation between tertiary-structure conservation and sequence identity between His-*Ov*MIF-1 and its structural neighbors (Supplementary Figs. S3, S4 and S5).

**Figure 3:**
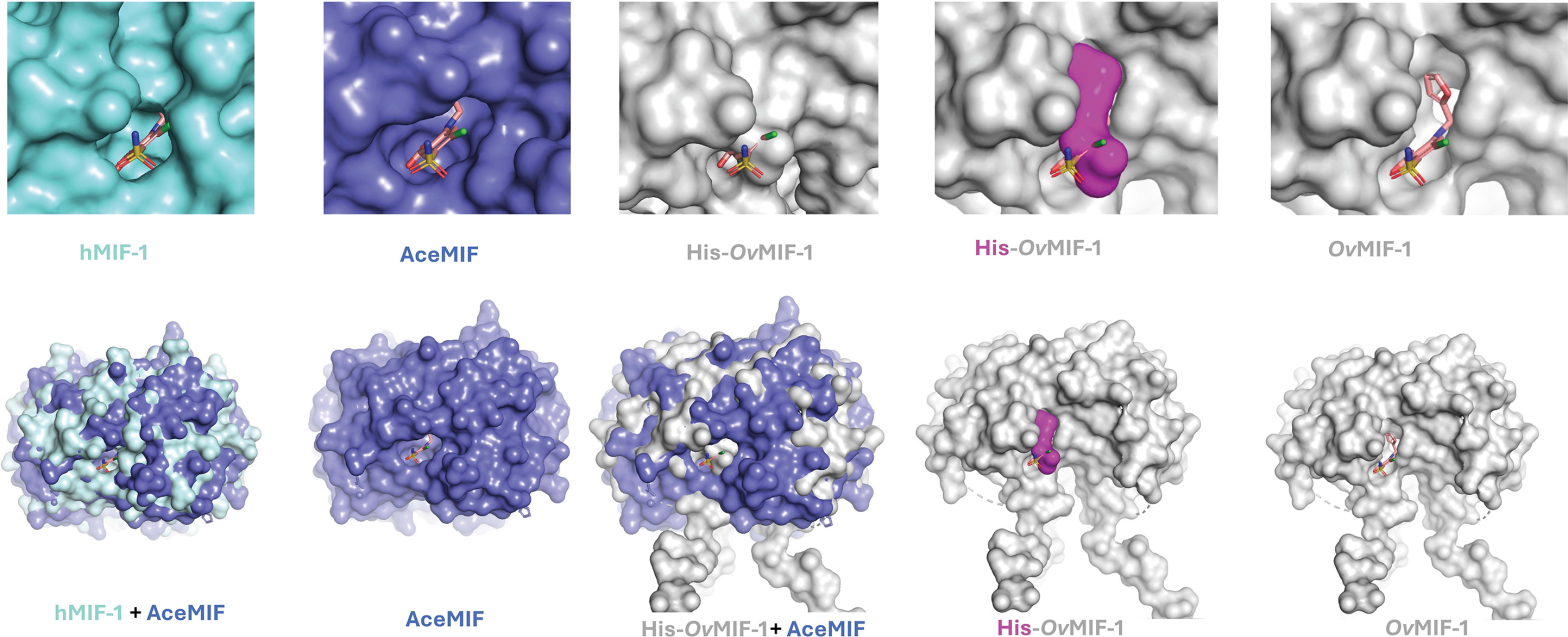
Comparison of tautomerase inhibitor-binding cavities in surface plots of hMIF-1 (cyan; PDB entry 1mif), *Ace*MIF (purple; PDB entry 3rf4) and His-*Ov*MIF-1 (gray). Furosemide (shown as sticks), a selective inhibitor of *Ace*MIF, fits best into its larger cavity of *Ace*MIF, hMIF has a smaller cavity and His-*Ov*MIF-1 lacks the cavity (top row). The superposed trimer surfaces are shown in the bottom row. The structure of His-*Ov*MIF-1 with the N-terminal tag in magenta is shown, as is the structure of *Ov*MIF-1 generated by deleting the tag. *Ov*MIF-1 has a more open cavity than the other structures.

One of the most-studied parasite MIF homologs is that from the minor human hookworm (*Ancylostoma ceylanicum*) targeted for drug development by Lolis and coworkers (Cho *et al.*, 2007 ▸, 2011 ▸). Like hMIF-1, the MIF homolog from *A. ceylanicum* (*Ace*MIF) binds the CD74 receptor, inhibits macrophage migration and has tautomerase activity; however, significant structural differences were observed in the tautomerase sites (Cho *et al.*, 2007 ▸). Interestingly, an hMIF-1 inhibitor, (*S*,*R*)-3-(4-hydroxyphenyl)-4,5-dihydro-5-isoxazole acetic acid, methyl ester (ISO-1), does not inhibit the tautomerase or chemoattractant activities of *Ace*MIF, and this difference in inhibition is likely to be due to structural differences in the enzymatic sites which allow the binding of ISO-1 by hMIF-1 but not by *Ace*MIF (Cho *et al.*, 2007 ▸). To identify structural differences that may be important for *Ace*MIF inhibition, we compared the active site of hMIF-1–ISO-1 with that of *Ace*MIF complexed with furosemide. This *Ace*MIF inhibitor was identified from drug-repositioning studies and from the Lolis group’s high-throughput screening of over 1000 FDA-approved drugs (Cho *et al.*, 2011 ▸). The structure of *Ace*MIF (PDB entry 3rf4, with furosemide) has an r.m.s.d. of 0.98 Å to *Ov*-MIF-1 on the alignment of 91 amino-acid residues, which is comparable to the r.m.s.d. of 1.12 Å for the alignment of hMIF-1 monomers (PDB entry 1mif). The tautomerase sites of hMIF-1–ISO-1 and *Ace*MIF–furosemide reveal a network of residues interacting with each ligand (Supplementary Fig. S5). These amino acids are all near the interaction between the N-terminus and the C-terminal loop that is abrogated by the presence of the N-terminal purification tag in our His-*Ov*MIF-1 structure. Thus, unlike the *Ace*MIF and hMIF-1 structures, the cavity of His-*Ov*MIF-1 for tautomerase inhibitors is blocked (Fig. 3 ▸).

**Figure 4:**
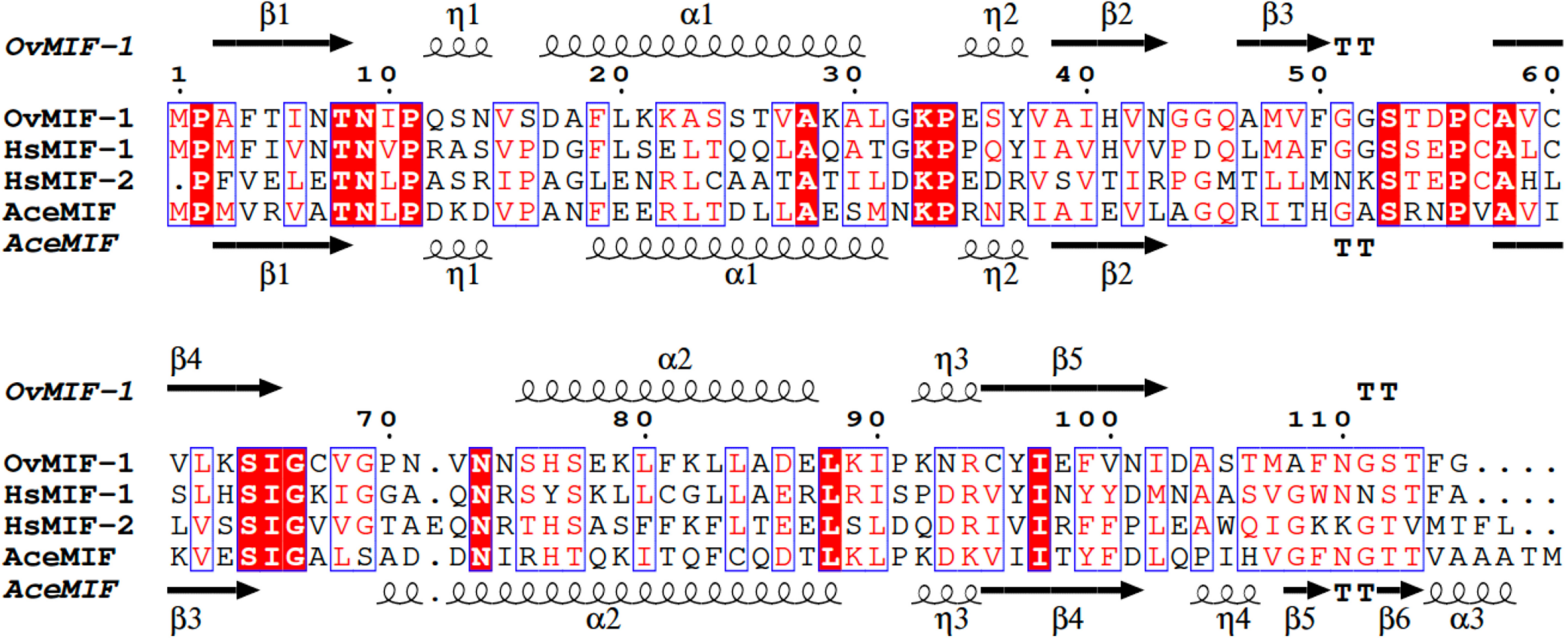
Structural and primary-sequence alignment of His-*Ov*MIF-1, hMIF-1, hMIF-2 and *Ace*MIF. The secondary-structure elements are as follows: α-helices are shown as large coils, 3_10_-helices are shown as small coils labeled h, β-strands are shown as arrows labeled β and β-turns are labeled TT. Identical residues are shown on a red background, with conserved residues in red and conserved regions in blue boxes. This figure was generated using *ESPript* 3.0 (Gouet *et al.*, 1999 ▸, 2003 ▸). Additional structural details and alignments are shown in Supplementary Fig. S2.

The amino-terminal proline of MIF acts as a catalytic base for phenylpyruvate tautomerase activity (Lubetsky *et al.*, 1999 ▸), which requires interactions with the carboxyl-terminal residues (Stamps *et al.*, 1998 ▸). While the proline is conserved in *Ov*MIF-1, its function is physically obscured in His-*Ov*MIF-1 by the additional N-terminal residues. Deleting the residues corresponding to the N-terminal extension in our structure exposes the cavity (Fig. 3 ▸), and *Ov*MIF-1 appears to possess all of the features necessary for tautomerase activity. Interestingly, the *in silico*-generated *Ov*MIF-1 cavity appears larger than that observed in hMIFs and easily accommodates furosemide, the selective *Ace*MIF inhibitor. Based on this observation and the sequence and structure conservation (Fig. 4 ▸), *Ace*MIF inhibitors may be suitable starting points for the design of *Ov*MIF-1 inhibitors. Other studies have shown that C-terminal histidine tags do not interfere with tautomerase, binding and other activities of plant MIFs (Sinitski *et al.*, 2020 ▸). Since the C-terminal residues of *Ov*MIF-1 do not block the tautomerase site, using a C-terminal tag for purification rather than an N-terminal tag to preserve the tautomerase activity of *Ov*MIF-1 is preferable.

Another binding partner of hMIF is the CD74 receptor (Leng *et al.*, 2003 ▸). hMIF–CD74 binding is unrelated to tautomerase binding, requires the formation of a trimer and is predicted to include the residues 50-FGGSEP-55, Lys76, 90-SPDR-93 and 109-NNS-111 from one monomer and residues 34-PQ-35, 108-WNN-110 and 111-STFA-114 from a second monomer (Meza-Romero *et al.*, 2016 ▸). These residues are partially conserved in *Ov*MIF-1 at the levels of conservation of *Ace*MIF (Fig. 4 ▸). *Ace*MIF is known to bind the CD74 receptor (Cho *et al.*, 2007 ▸). Further studies are required to determine whether *Ov*MIF-1 can interact with CD74. Additional studies are required to determine whether previously identified hMIF-1 allosteric inhibitors (Bai *et al.*, 2012 ▸; Cirillo *et al.*, 2020 ▸) will bind to *Ov*MIF-1.

## 4. Conclusion

We report the production, crystallization and 1.9 Å resolution structure of N-terminally hexahistidine-tagged *O. volvulus* macrophage migration inhibitory factor-1 (His-*Ov*MIF-1). The carboxyl-terminal residues of His-*Ov*MIF-1 form a long tail instead of folding back to interact with the N-terminus to form the tautomerase cavity. *In silico* removal of the N-terminal tag exposes a large cavity that can be used for drug discovery. Future studies will include testing the activity, removing the N-terminal tag and generating co-crystal structures of *Ov*MIF-1 with known MIF inhibitors.

## Supplementary Material

PDB reference: *Onchocerca volvulus* macrophage migration inhibitory factor-1, 8vj2

Supplementary Figures and Table. DOI: 10.1107/S2053230X24010550/ir5038sup1.pdf

## Figures and Tables

**Table 1:** Macromolecule-production information

Source organism	*Onchocerca volvulus*
DNA source	CollegeCodon optimized and synthetically generated plasmid from Twist Bioscience
Expression vector	pET-28a, AVA N-terminal tag
Expression host	*Escherichia coli* BL21(DE3) Rosetta
Complete amino-acid sequence of the construct produced†	MAHHHHHHMGTLEAQTQGPGSMPAFTINTNIPQSNVSDAFLKKASSTVAKALGKPESYVAIHVNGGQAMVFGGSTDPCAVCVLKSIG**CVGPNV**NNSHSEKLFKLLADELKIPKNRCYIEFVNIDASTMAFNGSTFG

† The N-terminal extension is underlined and the disordered loop residues are shown in bold and underlined.

**Table 2:** Crystallization

Method	Vapor diffusion, sitting drop
Plate type	Tray 101-d6, 96-well plates
Temperature (K)	291
Protein concentration (mg ml^−1^)	20.1
Buffer composition of protein solution	25 m*M* HEPES pH 7.0, 500 m*M* NaCl, 5% glycerol, 2 m*M* DTT, 0.025% azide
Composition of reservoir solution	100 m*M* bis-Tris–HCl pH 6.5, 400 m*M* sodium chloride, 30%(*w*/*v*) PEG 3350
Volume and ratio of drop	0.2 µl, 1:1
Volume of reservoir (µl)	50

**Table 3:** Data collection and processing Values in parentheses are for the outer shell.

Diffraction source	Beamline 19-ID, NSLS-II
Temperature (K)	100
Detector	Dectris EIGER2 XE 9M
Space group	*C*222_1_
*a*, *b*, *c* (Å)	52.44, 91.13, 132.49
α, β, γ (°)	90, 90, 90
Resolution range (Å)	45.45–1.90 (1.95–1.90)
Total No. of reflections	340455 (25834)
Completeness (%)	99.9 (99.2)
Multiplicity	13.4 (14.0)
〈*I*/σ(*I*)〉	15.0 (2.1)
*R*_r.i.m._	0.091 (1.136)
Overall *B* factor from Wilson plot (Å^2^)	50.48

**Table 4:** Structure refinement Values in parentheses are for the outer shell.

Resolution range (Å)	45.45–1.90 (1.98–1.90)
Completeness (%)	99.3
σ Cutoff	*F* > 1.34σ(*F*)
No. of reflections, working set	25255 (2549)
No. of reflections, test set	1249 (178)
Final *R*_cryst_	0.217 (0.3317)
Final *R*_free_	0.236 (0.4181)
No. of non-H atoms	
Protein	2398
Ion	1
Ligand	0
Water	83
Total	2482
R.m.s. deviations	
Bond lengths (Å)	0.004
Angles (°)	0.700
Average *B* factors (Å^2^)	
Protein	58.2
Ion	73.9
Ligand	0.0
Water	46.0
Ramachandran plot	
Most favored (%)	95.9
Allowed (%)	4.1
