# Supplementary material for: Crystal structure of N-terminally hexahistidine-tagged Onchocerca volvulus macrophage migration inhibitory factor-1
Source: Acta Crystallogr F Struct Biol Commun. 2024 Nov 6;80(Pt 12):328–34. doi: 10.1107/S2053230X24010550 (PMC11614107; doi:10.1107/S2053230X24010550)
Supplement: Supplementary file 1 [file f-80-00328-sup1.pdf]

STRUCTURAL BIOLOGY COMMUNICATIONS

Volume 80 (2024)

Supporting information for article:

Crystal structure of N-terminally hexahistidine-tagged *Onchocerca volvulus* macrophage migration inhibitory factor-1

Amber D. Kimble, Omolara C. O. Dawson, Lijun Liu, Sandhya Subramanian, Anne Cooper, Kevin Battaile, Justin Craig, Elizabeth Harmon, Peter Myler, Scott Lovell and Oluwatoyin A. Asojo

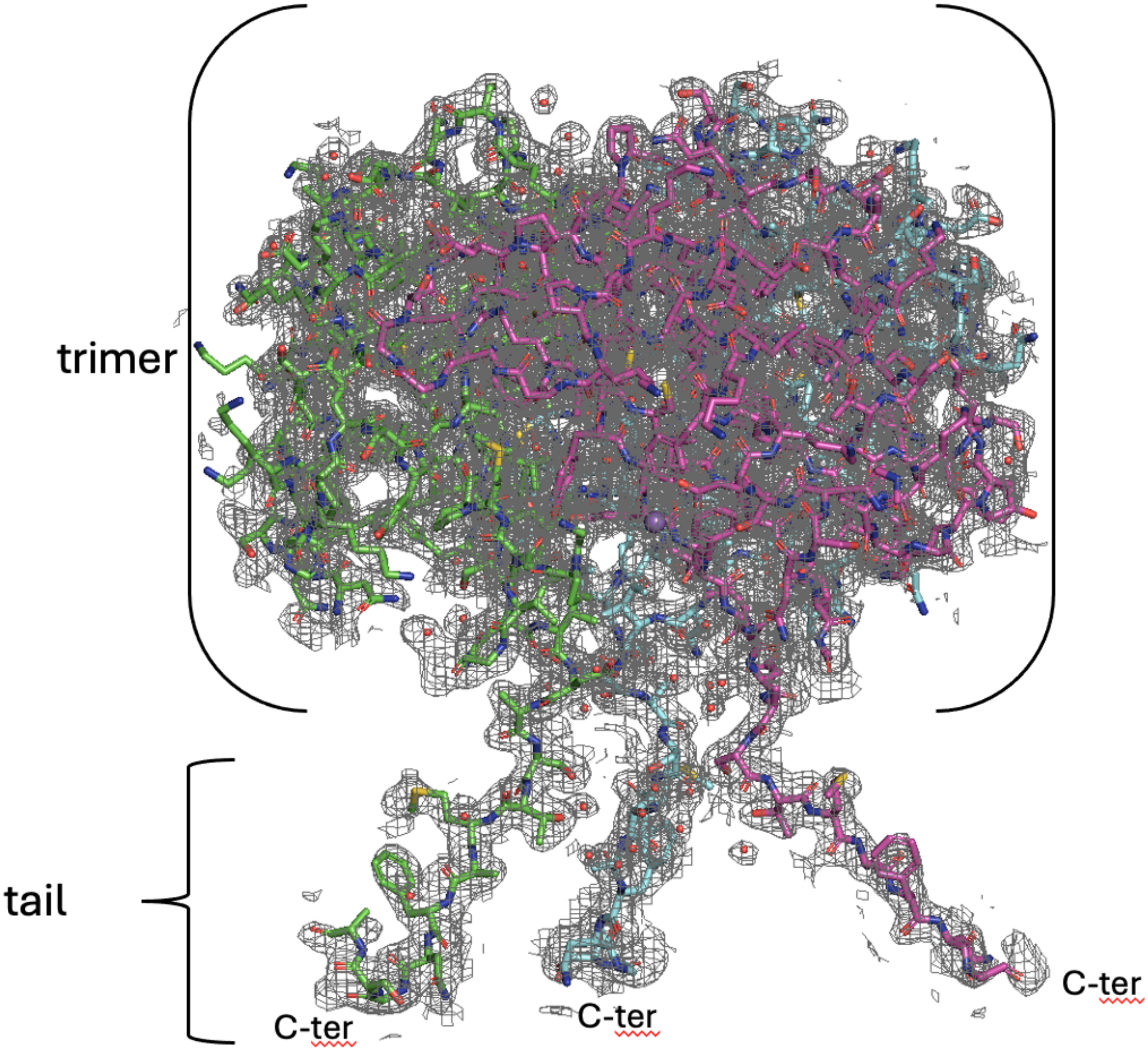

Figure S1

The His-*Ov*MIF-1 trimer in 2Fo-Fc map (gray mesh) contoured at 1.0 σ. The monomers are colored by elements (red for oxygen, green, and blue for nitrogen). Each monomer's main chain carbons are green, cyan, or magenta.

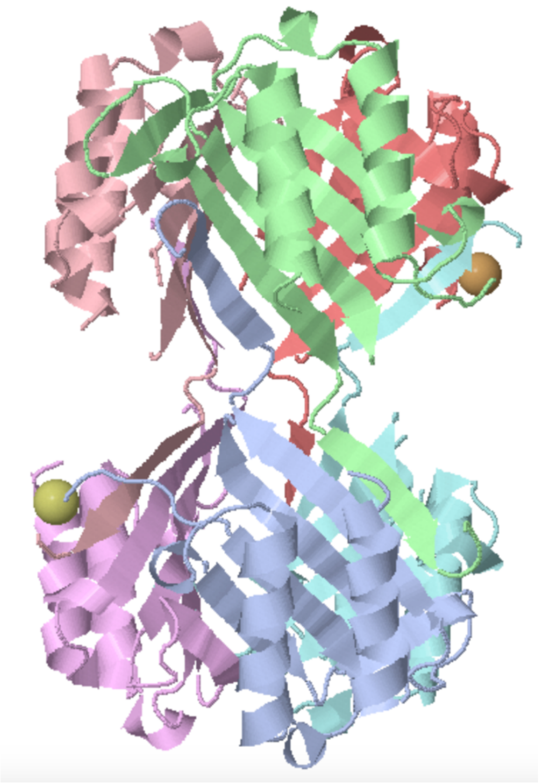

Analysis of protein interfaces suggests that the following quaternary structures are stable in solution.

Details | Download | View | XML

PQS set NN	«»	mm Size	Formula	Composition	Id	Biomol R350	Stable	Surface area, sq. Å	Buried area, sq. Å	ΔG^{int}, kcal/mol	ΔG^{diss}, kcal/mol
1	◉	6	A_6a_2	$A_2B_2C_2$[NA]$_2$	1	–	yes	25060	15460	-95.3	72.5
2	○	3	A_3a	ABC[NA]	2	1	yes	16350	3910	-22.6	11.5
3(*)	○	2	A_2a	BC[NA]	3	–	yes	11490	1810	-17.9	7.5
	○	2	A_2	A_2	3	–	yes	12090	1820	-9.0	4.8

Figure S2 PDBePISA (Proteins, Interfaces, Structures and Assemblies) analysis

The His-*Ov*MIF-1 hexamer is stabilized by three intermolecular beta-sheets formed by the three tails and symmetry-related monomers. The hexamer is considerably more stable than the trimer based on the AG^{diss} values.

'Protein interfaces, surfaces and assemblies' service PISA at the European Bioinformatics Institute. (http://www.ebi.ac.uk/pdbe/prot_int/pistart.html), E. Krissinel and K. Henrick (2007). *'Inference of macromolecular assemblies from crystalline state.'*. J. Mol. Biol. **372**, 774-79

E. Krissinel (2009). *Crystal contacts as nature's docking solutions*. J Comput Chem. 2010 Jan 15;31(1):133-43.; DOI 10.1002/jcc.21303

E. Krissinel and K. Henrick (2005). *Detection of Protein Assemblies in Crystals*. In: M.R. Berthold *et.al.* (Eds.): CompLife 2005, LNBI 3695, pp. 163--174. Springer-Verlag Berlin Heidelberg.

Table S1 PDBeFold Top 49 results

PDBe Fold v2.59. (src3) 14 Apr 2014 result file.

RESULT SUMMARY

##	Q-score	P-score	Z-score	RMSD	Nalgn	Nsse	Ngaps	Seq-%	Nmd	Nres-Q	Nsse-Q	Nres-T	Nsse-T	Query	Target
1	0.9643	3.89	5.838	0.000	109	7	1	1	0	111	8	111	9	PDB 8vj2:A	PDB 7ms3:A
2	0.9643	2.846	5.09	0.000	109	6	1	1	0	111	8	111	7	PDB 8vj2:A	PDB 7ms9:A
3	0.9643	3.89	5.838	0.000	109	7	1	1	0	111	8	111	9	PDB 8vj2:A	PDB 7ms1:A
4	0.9643	3.76	5.75	0.000	109	7	1	1	0	111	8	111	9	PDB 8vj2:A	PDB 7ms8:A
5	0.8866	3.261	5.423	0.579	105	7	2	1	0	111	8	108	10	PDB 8vj2:A	PDB 7ms1:B
6	0.855	2.207	4.565	0.763	103	6	2	1	0	111	8	105	7	PDB 8vj2:A	PDB 7ms9:C
7	0.7087	13.85	11.01	1.112	96	8	1	0.4375	0	111	8	103	8	PDB 8vj2:A	PDB 1fim:A
8	0.705	11.97	10.34	0.921	93	7	2	0.4516	0	111	8	101	8	PDB 8vj2:A	PDB 4gum:H
9	0.6558	12	10.35	1.056	94	7	2	0.4574	0	111	8	108	8	PDB 8vj2:A	PDB 4gum:F
10	0.6431	12.25	10.46	1.062	94	7	2	0.4574	0	111	8	110	8	PDB 8vj2:A	PDB 4gum:D
11	0.6393	12.52	10.52	1.003	94	7	2	0.4574	0	111	8	112	9	PDB 8vj2:A	PDB 4gum:C
12	0.6354	12.2	10.38	0.989	94	7	2	0.4574	0	111	8	113	8	PDB 8vj2:A	PDB 4gum:I
13	0.6318	12.28	10.47	1.211	96	7	1	0.5312	0	111	8	113	9	PDB 8vj2:A	PDB 1hfo:C
14	0.6271	14.61	11.44	1.010	94	8	2	0.4574	0	111	8	114	9	PDB 8vj2:A	PDB 4gum:A
15	0.6255	11.39	10.08	1.123	95	7	1	0.4632	0	111	8	114	9	PDB 8vj2:A	PDB 6bg7:B
16	0.6252	11.32	10.05	1.180	96	7	1	0.4688	0	111	8	115	9	PDB 8vj2:A	PDB 1gif:A
17	0.6248	11.49	10.12	1.128	95	7	1	0.4632	0	111	8	114	9	PDB 8vj2:A	PDB 5b4o:A
18	0.6248	12.21	10.44	1.258	96	7	1	0.5312	0	111	8	113	9	PDB 8vj2:A	PDB 1hfo:D
19	0.6244	12.43	10.54	1.131	95	7	2	0.4632	0	111	8	114	9	PDB 8vj2:A	PDB 6fve:A
20	0.6241	11.39	10.08	1.134	95	7	1	0.4526	0	111	8	114	9	PDB 8vj2:A	PDB 5bsj:A
21	0.6238	11.39	10.08	1.136	95	7	1	0.4632	0	111	8	114	9	PDB 8vj2:A	PDB 4p0h:B
22	0.6236	11.39	10.08	1.137	95	7	1	0.4632	0	111	8	114	9	PDB 8vj2:A	PDB 5xej:B
23	0.6235	11.22	10.01	1.178	95	7	1	0.4632	0	111	8	113	9	PDB 8vj2:A	PDB 6bg7:A
24	0.6234	11.32	10.05	1.138	95	7	1	0.4632	0	111	8	114	9	PDB 8vj2:A	PDB 2ooh:A
25	0.6231	11.59	10.17	1.084	94	7	1	0.5213	0	111	8	113	9	PDB 8vj2:A	PDB 1hfo:A
26	0.6228	11.9	10.31	1.183	95	7	2	0.5474	0	111	8	113	9	PDB 8vj2:A	PDB 1hfo:B
27	0.6223	11.29	10.04	1.146	95	7	1	0.4632	0	111	8	114	9	PDB 8vj2:A	PDB 6cb5:B
28	0.6222	11.22	10.01	1.186	95	7	1	0.4526	0	111	8	113	9	PDB 8vj2:A	PDB 5bsj:C
29	0.6219	11.19	9.99	1.149	95	7	1	0.4632	0	111	8	114	9	PDB 8vj2:A	PDB 4wr8:F
30	0.6216	11.32	10.05	1.205	96	7	1	0.4688	0	111	8	115	9	PDB 8vj2:A	PDB 1gif:B
31	0.6214	11.32	10.05	1.153	95	7	1	0.4632	0	111	8	114	9	PDB 8vj2:A	PDB 5j7p:B
32	0.6213	11.29	10.04	1.154	95	7	1	0.4632	0	111	8	114	9	PDB 8vj2:A	PDB 6b1c:A
33	0.6212	11.25	10.02	1.154	95	7	1	0.4632	0	111	8	114	8	PDB 8vj2:A	PDB 4wr8:R
34	0.6211	10.32	9.591	1.194	95	7	1	0.4526	0	111	8	113	9	PDB 8vj2:A	PDB 4z1t:B
35	0.621	11.83	10.28	1.156	95	7	1	0.4632	0	111	8	114	9	PDB 8vj2:A	PDB 4p01:B
36	0.6209	11.15	9.975	1.157	95	7	1	0.4632	0	111	8	114	9	PDB 8vj2:A	PDB 6b2c:A
37	0.6208	11.29	10.04	1.211	96	7	1	0.4688	0	111	8	115	9	PDB 8vj2:A	PDB 1gif:C
38	0.6207	11.32	10.05	1.158	95	7	1	0.4526	0	111	8	114	9	PDB 8vj2:A	PDB 1p1g:A
39	0.6206	9.794	9.343	1.212	96	7	1	0.4479	0	111	8	115	9	PDB 8vj2:A	PDB 1cgq:A
40	0.6206	9.824	9.358	1.213	96	7	1	0.4479	0	111	8	115	9	PDB 8vj2:A	PDB 1cgq:C
41	0.6205	13.61	10.91	1.160	95	8	1	0.4526	0	111	8	114	8	PDB 8vj2:A	PDB 5bsi:G
42	0.6205	11.22	10.01	1.160	95	7	1	0.4632	0	111	8	114	9	PDB 8vj2:A	PDB 4wr8:G
43	0.6204	11.25	10.02	1.161	95	7	1	0.4632	0	111	8	114	9	PDB 8vj2:A	PDB 5umj:B
44	0.6203	9.794	9.343	1.214	96	7	1	0.4479	0	111	8	115	9	PDB 8vj2:A	PDB 1cgq:B
45	0.6203	11.22	10.01	1.161	95	7	1	0.4632	0	111	8	114	9	PDB 8vj2:A	PDB 4oyq:C
46	0.6202	11.29	10.04	1.162	95	7	1	0.4316	0	111	8	114	9	PDB 8vj2:A	PDB 1mfi:A
47	0.6201	11.29	10.04	1.163	95	7	1	0.4526	0	111	8	114	9	PDB 8vj2:A	PDB 5bsi:E
48	0.62	11.29	10.04	1.163	95	7	1	0.4526	0	111	8	114	9	PDB 8vj2:A	PDB 5bsi:C
49	0.62	11.29	10.04	1.163	95	7	1	0.4526	0	111	8	114	9	PDB 8vj2:A	PDB 1p1g:B

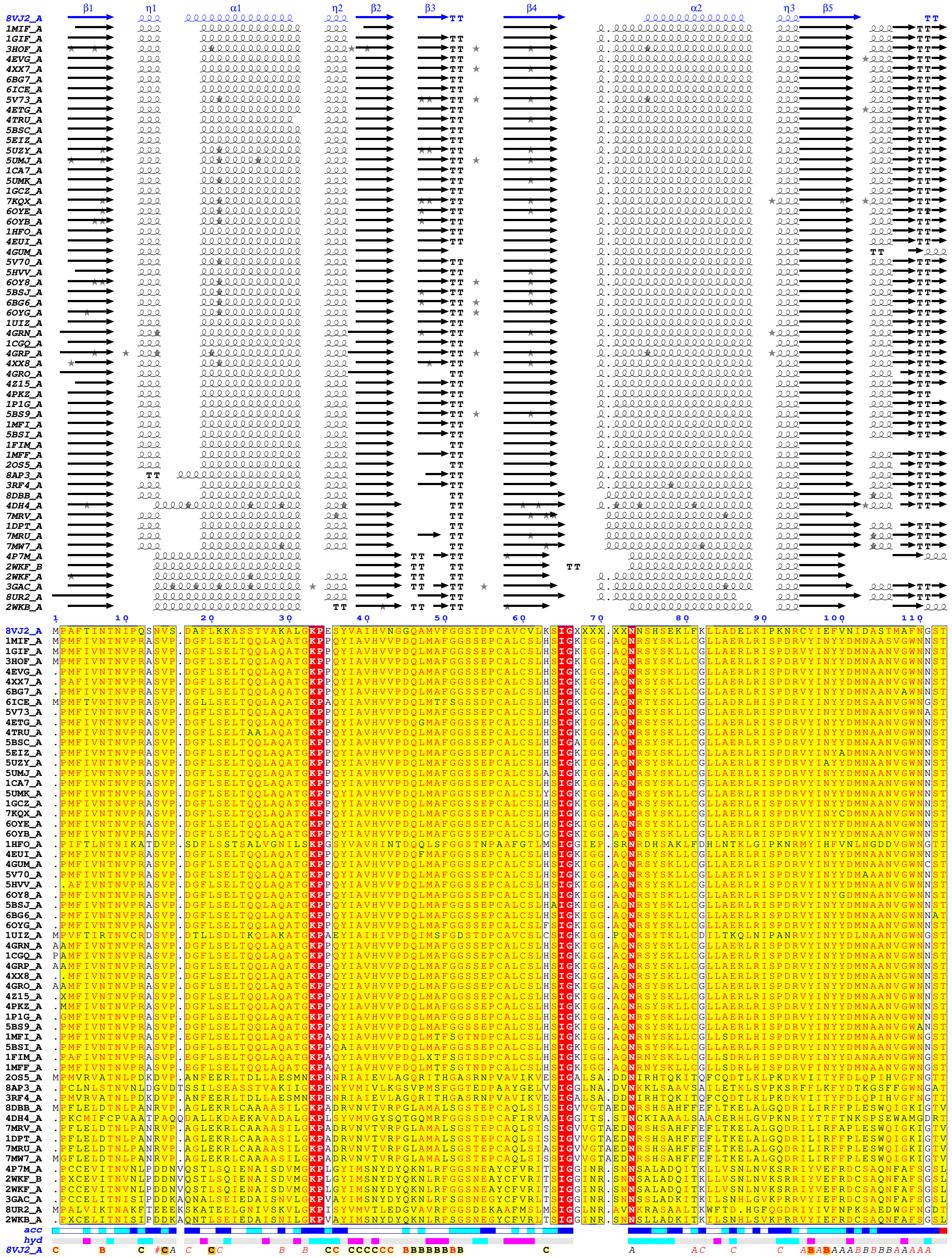

Figure S3

ENDSCRIPT reveals the nearest structural neighbors of His-*Ov*MIF-1. Identical and conserved residues are highlighted in red and yellow, respectively. The different secondary structure elements shown are alpha helices (α), 3_{10}-helices (η), beta strands (β), and beta turns (TT).

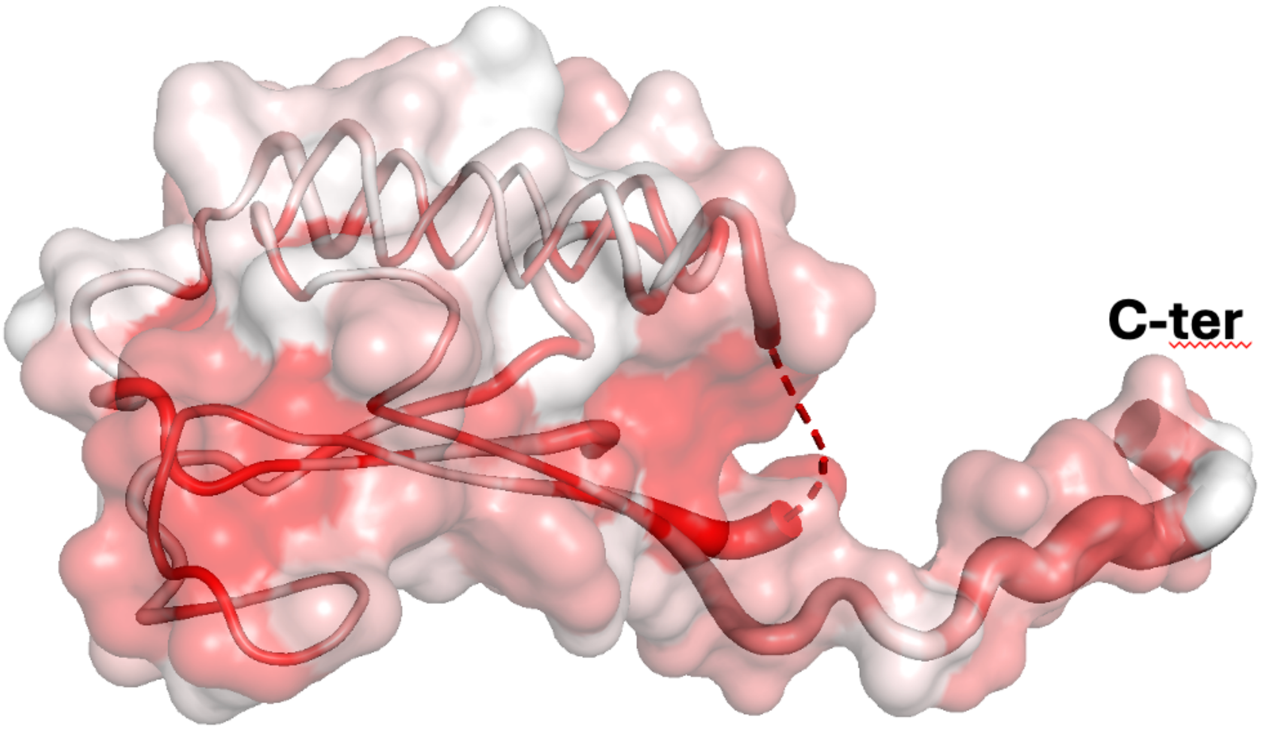

Figure S4

Ribbon diagram calculated by ENDScript. Circumference of the ribbon (sausage) represents relative structural conservation compared to other MIF structures (these structures are indicated in Figure S.2). Thinner ribbons represent higher conserved regions. In comparison, thicker ribbons represent lower conserved regions, and the ribbon is colored by sequence conservation, with red indicating identical residues. The 50% transparent surface is also shown.

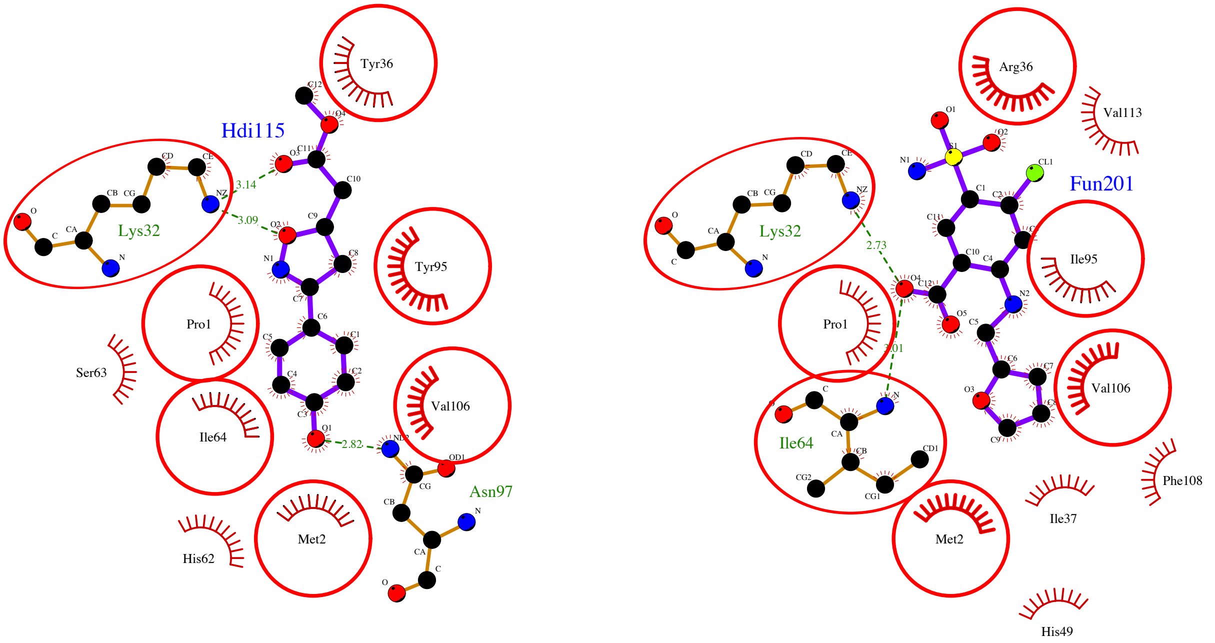

Figure S5

The active sites of hMIF with ISO-1 (Hdi115A) and AceMIF with furosemide (Fun201) reveal a network of interactions from two monomers in both structures. PDB codes are 3RF4 (AceMIF/furosemide) and 1LJT (hMIF/ISO-1).
